# *In vitro* virucidal activity of a commercial disinfectant against viruses of domestic animals and poultry

**DOI:** 10.3389/fvets.2023.1276031

**Published:** 2024-01-04

**Authors:** Nader M. Sobhy, Angie Quinonez-Munoz, Hamada A. Aboubakr, Christiana R. B. Youssef, Gonzalo Ojeda-Barría, Jonathan Mendoza-Fernández, Sagar M. Goyal

**Affiliations:** ^1^Department of Veterinary Population Medicine, University of Minnesota, Saint Paul, MN, United States; ^2^Department of Animal Medicine, Faculty of Veterinary Medicine, Zagazig University, Zagazig, Sharkia, Egypt; ^3^Department of Microbiology and Immunology, Faculty of Pharmacy, Zagazig University, Zagazig, Egypt; ^4^Veterquimica S.A., Santiago, Chile

**Keywords:** Duplalim, disinfection, virus, inactivation, suspension test

## Abstract

Outbreaks of viral diseases in animals are a cause of concern for animal welfare and economics of animal production. One way to disrupt the cycle of infection is by combating viruses in the environment and prohibiting them from being transmitted to a new host. Viral contamination of the environment can be reduced using well-tested and efficacious disinfectants. Duplalim is a commercially available disinfectant consisting of 12% glutaraldehyde and 10% quaternary ammonium compounds. We evaluated this disinfectant for its efficacy against several viruses in poultry (*n* = 3), pigs (*n* = 5), dogs (*n* = 2), and cattle (*n* = 4). In suspension tests, 1:100 dilution of Duplalim was found to inactivate more than 99% of these 14 viruses in 15 min or less. The titers of a majority of these viruses decreased by ≥99.99% in <60 min of contact time. In conclusion, the ingredient combination in Duplalim is very effective in inactivating common viruses of domestic animals and poultry.

## Introduction

Viral diseases are problematic not only for public health but also cause huge economic losses to livestock and poultry industries. The recent pandemic of SARS-CoV-2, the causative agent of COVID-19, underscored once again the hazards associated with viral pathogens. Outbreaks of viral diseases in domestic animals and poultry result in huge economic losses due to their sudden onset, rapid spread, and even death. For example, the economic burden of infectious bovine rhinotracheitis virus (IBRV) in cattle is estimated to be $1.5 to $2.5 billion per year. The outbreaks of avian influenza in 2015 resulted in loss of $1.7 billion (1). Hence, the management and control of these diseases is important in terms of economics as well as to avoid food security crisis. Viral diseases in companion animals, e.g., those caused by canine parvovirus and canine distemper virus, are emotionally damaging as well as costly to treat and control.

Viruses are transmitted from one host to the other by direct and indirect routes. For the indirect route to be successful, the viruses that are shed in excretions and secretions of infected hosts may contaminate the environment including inanimate fomites and surfaces. Naïve hosts may be infected with viruses when they encounter virus-contaminated objects (2). Cleaning and disinfection of the environment on animal farms and kennels are undertaken to inactivate viruses, if present. An ideal disinfectant should inactivate the viruses rapidly and be safe for the environment.

Before a new disinfectant is placed in use, it is necessary to demonstrate its efficacy. Indiscriminate use of non-effective disinfectants can lead to unnecessary environmental contamination and may help increase the appearance of resistant strains of pathogens (3). Hence, it is important to evaluate their efficacy since the use of an appropriate disinfectant can limit virus spread and minimize economic losses. One of the widely used disinfectants is Duplalim® (Veterquímica S.A., Chile), which is formulated with 12% glutaraldehyde (GLT) and 10% quaternary ammonium mixture (7% benzalkonium and 3% other quaternary ammonium compounds). The QACs in Duplalim are cationic surfactants that are non-toxic and highly tolerant to the presence of organic matter (4). Tsujimura et al. (5) demonstrated that the addition of 5% fetal bovine serum (FBS) as an organic compound did not change the virucidal effect of didecyl dimethyl ammonium chloride (DDC) (5). However, it did increase the virucidal power of BZK by four times. GLT, widely used in hospitals, is a broad-spectrum sterilizing and disinfecting agent, which can act within a short period of exposure (6, 7).

According to the U.S. Environmental Protection Agency, a disinfectant must be evaluated against each class of pathogen against which it is to be used. A viral disinfectant must show inactivation of 2.8 to 4 log_10_ of the virus (8). This study was performed to investigate the *in vitro* effectiveness of a commercial, broad-spectrum disinfectant (Duplalim) against common viral pathogens of bovine, porcine, canine, and avian species. Duplalim consists of 12% glutaraldehide and a 10% mix of quaternary ammonium compounds (7% benzalkonium and 3% other).

## Materials and methods

### Test viruses

Common, economically important viruses of various hosts (avian, porcine, bovine, and canine) were used in this study. A variety of viruses were included, e.g., enveloped, and non-enveloped viruses, and viruses with single stranded RNA, double stranded RNA, single stranded DNA, and double stranded DNA. For poultry, chicken reovirus (CRV), fowl adenovirus [the causative agent of inclusion body hepatitis (IBH)], and Newcastle disease virus (NDV) were selected. For swine, Seneca virus A (SVV), and two subtypes of swine influenza virus (H1N1 and H3N2) were used. Viruses affecting dogs were canine distemper virus (CDV) and canine parvovirus (CPV). Bovine viruses included infectious bovine rhinotracheitis virus (IBR), bovine viral diarrhea virus (BVDV), bovine respiratory syncytial virus (BRSV), and bovine coronavirus (BCV). All viruses were propagated and titrated in their specific, susceptible cells (Table 1). After propagation, stock viruses were aliquoted in 1 mL amounts and stored at−80°C. On the day of use, an aliquot was removed, thawed, and placed on ice until used in the experiment.

**Table 1:** Viruses and cell lines used in the study.

Virus	Characteristics	Cells used
Chicken reovirus	Non-enveloped, double stranded RNA	QT-35 (quail turbinate)
Chicken adenovirus	Non-enveloped, double stranded DNA	Vero-76 (African Green monkey kidney)
Newcastle disease virus	Enveloped, single stranded RNA	LLC-MK2 (Rhesus monkey kidney)
Porcine Senecavirus A	Non-enveloped, single stranded RNA	ST (swine testicular)
Swine influenza virus subtypes H1N1 and H3N2	Enveloped, single stranded RNA	MDCK (Madin-Darby canine kidney)
Canine distemper virus	Enveloped, single stranded RNA	Vero-76
Canine parvovirus	Non-enveloped, single stranded DNA	CRFK (Crandell-Reese feline kidney)
Infectious bovine rhinotracheitis virus	Enveloped, double stranded DNA	MDBK (Madin-Darby bovine kidney)
Bovine viral diarrhea virus	Enveloped, single stranded RNA	MDBK
Bovine respiratory syncytial virus	Enveloped, single stranded RNA	MDBK
Bovine coronavirus	Enveloped, single stranded RNA	MDBK

### Cell cultures

Cell lines exhibiting cytopathic effects (CPE) upon viral infection were used for virus propagation and titration. All cells (Table 1) were grown in Eagle’s minimum essential medium (MEM) containing penicillin 150 IU/mL, streptomycin 150 μg/mL, neomycin 50 μg/mL, ciprofloxacin 10 μg /ml, and fungizone 1.5 μg/mL with 8% fetal bovine serum (FBS; or donor horse serum when testing bovine viruses). The cells were maintained and used as monolayers in disposable tissue culture flasks and 96-well microtiter plates as needed. On the day of testing, the cells were examined to ensure that they had proper cell integrity and were suitable for virus titrations.

### Suspension test

Different dilutions of Duplalim were prepared in MEM (Tables 2–5). To 500 μL of each dilution was added an equal amount (500 μL) of the test virus. As negative controls, 500 μL of MEM was mixed with an equal amount (500 μL) of a given virus but no disinfectant was added. Samples of each mixture were withdrawn at different intervals of time. The used dilutions and duration of exposure are shown in Tables 2–5. Serial 10-fold dilutions of the samples, obtained at different time points, were prepared in MEM followed by inoculation in monolayers of appropriate cell cultures contained in 96-well microtiter plates (Table 1). Triplicate wells were used for all dilutions and the inoculum size for each well was 100 μL. The plates were incubated at 37°C under 5% CO_2_ for 90 min (time for virus attachment to cells) followed by washing twice with Hanks’ balanced salt solution (HBSS) to minimize cytotoxicity of Duplalim. Fresh MEM was then added to all wells at 100 μL per well. The plates were re-incubated at 37°C for up to 7 days and were examined daily under an inverted microscope for the appearance of viral-induced cytopathic effects (CPE). Virus titers were calculated by the Karber method and expressed as 50% tissue culture infective dose (TCID_50_) (9). Percent virus inactivation at each time point was calculated by comparing virus titers in Duplalim-treated versus negative control at each time point.

**Table 2:** The effect of Duplalim on avian viruses.

Virus (initial titer)	Final Duplalim dilution	Time (min)	Titer (Log_10_ TCID_50_/0.1 mL)		Virus reduction (%)
			Control	Duplalim-treated	
Chicken reovirus (4.83 TCID_50_/0.1 mL)	1:100	5	4.83	≤0.50	≥99.99
		15	4.50	≤0.50	≥99.99
		30	4.61	≤0.50	≥99.99
		60	4.61	≤0.50	≥99.99
	1:200	5	4.83	4.50	53.58
		15	4.50	≤0.50	≥99.99
		30	4.61	≤0.50	≥99.99
		60	4.61	≤0.50	≥99.99
	1:400	5	4.83	4.61	39.74
		15	4.50	4.39	22.96
		30	4.61	3.83	83.40
		60	4.61	3.72	87.11
Inclusion body hepatitis (4.67 TCID_50_/0.1 mL)	1:100	5	4.67	0.50	≥99.99
		15	4.67	0.50	≥99.99
		30	NT^*^	NT	NT
		60	NT	NT	NT
	1:200	5	4.67	3.34	95.32
		15	4.67	0.50	≥99.99
		30	NT	NT	NT
		60	NT	NT	NT
	1:400	5	4.67	3.83	85.37
		15	4.67	3.34	95.32
		30	4.67	2.67	99.00
		60	4.67	1.83	99.85
Newcastle disease virus (7.17 TCID_50_/0.1 mL)	1:100	5	7.28	5.06	99.40
		15	7.05	3.50	99.97
		30	7.17	2.95	99.99
		60	7.28	2.17	99.99
	1:200	5	7.28	6.28	90.07
		10	7.05	4.83	99.39
		30	7.17	4.50	99.78
		60	7.28	3.50	99.98
	1:400	5	7.28	6.39	87.11
		15	7.05	5.83	93.97
		30	7.17	5.95	94.02
		60	7.28	6.06	93.97

*NT = not tested.

**Table 3:** The effect of Duplalim on porcine viruses.

Virus (initial titer)	Final Duplalim dilution	Time (min)	Virus Titer (Log_10_ TCID_50_/0.1 mL)		Virus reduction (%)
			Control	Duplalim-treated	
Seneca valley virus (7.17 TCID50/0.1 mL)	1:100	5	7.17	6.39	83.40
		15	7.06	4.28	99.83
		30	7.28	3.50	99.98
		60	7.17	2.83	99.99
	1:200	5	7.17	6.50	78.62
		10	7.06	5.50	97.22
		30	7.28	5.39	98.71
		60	7.17	5.28	98.70
	1:400	5	7.17	7.06	22.96
		15	7.06	6.61	64.24
		30	7.28	6.50	83.40
		60	7.17	6.28	87.01
Swine influenza (H1N1) (5.17 TCID_50_/0.1 mL)	1:100	5	5.17	≤0.50	≥99.99
		15	5.17	≤0.50	≥99.99
		30	4.94	≤0.50	≥99.99
		60	5.06	≤0.50	≥99.99
	1:200	5	5.17	≤0.50	≥99.99
		15	5.17	≤0.50	≥99.99
		30	4.94	≤0.50	≥99.99
		60	5.06	≤0.50	≥99.99
	1:400	5	5.17	≤0.50	≥99.99
		15	5.17	≤0.50	≥99.99
		30	4.94	≤0.50	≥99.99
		60	5.06	≤0.50	≥99.99
Swine influenza (H3N2) (4.61TCID_50_/0.1 mL)	1:100	5	4.94	≤0.50	≥99.99
		15	4.83	≤0.50	≥99.99
		30	4.83	≤0.50	≥99.99
		60	4.72	≤0.50	≥99.99
	1:200	5	4.94	≤0.50	≥99.99
		15	4.83	≤0.50	≥99.99
		30	4.83	≤0.50	≥99.99
		60	4.72	≤0.50	≥99.99
	1:400	5	4.94	≤0.50	≥99.99
		15	4.83	≤0.50	≥99.99
		30	4.83	≤0.50	≥99.99
		60	4.72	≤0.50	≥99.99

**Table 4:** The effect of Duplalim on dogs viruses.

Virus (initial titer)	Final Duplalim dilution	Time (min)	Titer (Log_10_ TCID_50_/0.1 mL)		Virus reduction (%)
			Control	Duplalim-treated	
Canine distemper virus (4.83TCID_50_/0.1 mL)	1:100	5	4.83	2.50	99.53
		15	4.83	1.50	99.95
		30	4.83	1.16	99.97
		60	4.83	0.83	99.99
	1:200	5	4.83	2.80	99.00
		15	4.83	1.83	99.90
		30	4.83	1.50	99.95
		60	4.83	1.16	99.97
Canine parvovirus (4.67 TCID_50_/0.1 mL)	1:100	5	4.50	3.20	94.98
		15	4.50	2.60	98.74
		30	4.50	2.30	99.37
		60	4.50	2.10	99.60
	1:200	5	4.50	3.50	90.00
		10	4.50	3.10	96.02
		30	4.50	2.50	99.00
		60	4.50	2.30	99.37

**Table 5:** The effect of Duplalim on bovine viruses.

Virus (initial titer)	Final Duplalim dilution	Time (min)	Virus titer (Log_10_ TCID_50_/0.1 mL)		Virus reduction (%)
			Control	Duplalim-treated	
Infectious bovine rhinotracheitis virus (6.28 TCID_50_/0.1 mL)	1:100 and 1:200	5	6.06	≤0.5	≥99.99
		15	6.50	≤0.5	≥99.99
		30	6.50	≤0.5	≥99.99
		60	6.50	≤0.5	≥99.99
Bovine viral diarrhea virus (4.17 TCID_50_/0.1 mL)	1:100 and 1:200	5	3.83	≤0.5	≥99.95
		15	4.50	≤0.5	≥99.99
		30	4.83	≤0.5	≥99.99
		60	4.50	≤0.5	≥99.99
Bovine respiratory syncytial virus (3.50 TCID_50_/0.1 mL)	1:100 and 1:200	5	3.50	≤0.50	≥99.90
		15	3.17	≤0.50	≥99.78
		30	3.50	≤0.50	≥99.90
		60	3.17	≤0.50	≥99.78
Bovine coronavirus (4.95TCID_50_/0.1 mL)	1:100 and 1:200	5	4.95	≤0.50	≥99.99
		15	4.39	≤0.50	≥99.98
		30	4.50	≤0.50	≥99.99
		60	4.50	≤0.50	≥99.99

## Results

### Avian viruses

The results of inactivation of avian viruses by Duplalim are shown in Table 2; Duplalim was effective against both IBH and CRV. The titers of both viruses decreased by ≥4 logs (≥99.99% inactivation) within 5 min at 1:100 dilution and within 15 min at 1:200 dilution. The NDV was slightly more resistant; it took 30 min for 1:100 dilution to inactivate ≥4 logs of this virus. At 1:200 dilution, Duplalim reduced the NDV titer by 2 and 3 logs within 15 and 60 min, respectively. At 1:400 dilution, only a 2log reduction was seen in IBH virus titer after 30 min (Table 2).

### Swine viruses

Both subtypes of SIV were highly susceptible to the action of Duplalim; ≥4 logs were inactivated at all three dilutions within 5 min of contact. Seneca A virus was less susceptible; only 3 logs of this virus was inactivated at 1:100 dilution within 30 min (Table 3).

### Canine viruses

At 1:100 and 1:200 dilutions, Duplalim inactivated 3 logs (99.9%) of CDV within 15 min. At 1:100 dilution, 4 logs of CDV were iactivated within 60 min. The CPV was slightly more resistant; 2 logs of this virus were killed within 30 min at 1:100 and 1:200 dilutions (Table 4).

### Bovine viruses

Duplalim killed ≥4 logs of IBRV and BCV (≥ 99.99%) within 5 min at 1:200 dilution. More than 3 logs of BVDV and BRSV were inactivated within 5 min at 1:200 dilution (Table 5).

## Discussion

The selection of an effective disinfectant against bacterial and viral pathogens is key to the success of any biosecurity program. In this study, Duplalim was able to inactivate all viruses tested within a short contact time. This is possibly because of the combination of glutaraldehyde and quaternary ammonium compounds in this disinfectant. In general, a combination of disinfectants is known to have high efficiency and broad-spectrum action against viruses (10, 11). For example, Mor et al. (12) showed that a combination of QACs and aldehyde performed better than phenol compounds to eliminate CRV. In another study, QAC alone failed to inactivate non-enveloped viruses (13). Fowl adenovirus (FAdV), a non-enveloped virus, resists phenol and QACs but is sensitive to GLT (14). However, Ruano et al. (15) noted that FAdV was resistant to 0.1% GLT when used alone.

Of avian viruses, Duplalim killed ≥99.99% (4 logs) of CRV and IBH at 1:100 dilution within 5 min. These results are compatible with those of Mor et al. (12) who showed that a combination of QACs and aldehyde could inactivate CRV. Duplalim killed more than 99.99% (4 logs) of the IBH virus within 5 min at 1:100 final dilution. However, NDV was a little more resistant; a 1:100 dilution of Duplalim caused 4 log reduction in NDV titer after 30 min. This is in contrast to a previous study in which Patnayak et al. (16) showed that 2.6% GLT was able to inactivate NDV almost instantaneously while QACs could not. After 1 min of exposure, GLT and QACS (0.5%) cause a 2.7 log titer reduction on cement and rubber, respectively, according to Gamal et al. (17). Moreover, Ito et al. note that QAC (x500) can inactivate NDV within 30 s in the absence or presence of 5% FBS (18). The contrasting results of these studies are not surprising; Nemoto et al. (19) have noted variations in GLT disinfection power at low and high temperatures.

Two different SIV strains were used in this study since different subtypes of influenza viruses show extensive variations in the glycophospholipids of their envelopes (20, 21). Duplalim easily inactivated both subtypes. This is not surprising; Rhee et al. (22) have shown that the ingredients in Duplalim are powerful against influenza viruses even when used individually. In addition, GLT has been shown to inactivate influenza viruses in previous studies (23, 24). Seneca virus was a bit more resistant to the action of Duplalim; 1:100 and 1:200 dilutions were able to inactivate 99.99 and 98.70% of this virus, respectively, but only after a contact time of 60 min. This is not surprising because non-enveloped viruses are known to exhibit greater resistance to commonly used disinfectants than enveloped viruses.

As far as canine viruses are concerned, 99% of CDV was inactivated within 5 min at 1:100 and 1:200 dilutions. At a contact time of 60 min, 1:100 and 1:200 dilutions were able to inactivate 99.99 and 99.97% of CDV, respectively. In contrast, Duplalim killed only 99% (2 logs) of CPV within 30 min at 1:100 and 1:200 dilutions. The reaction time for CPV inactivation increased probably because parvoviruses are known to be more persistent in the environment and resist most disinfectants (25). The observed inactivation of CPV, albeit at a low level in this study, is probably because of the combined effect of QAC and GLT. The individual use of GLT, QACs, and GLT-based products showed poor results against certain parvoviruses, e.g., porcine parvovirus (PPV) and minute virus of mice (26) although 2% GLT has been found effective against MVM and PPV (27, 28).

Duplalim was able to inactivate ≥99.99% of IBRV and ≥ 99.9% of BVDV within 5 min. A Mixture of QACs and GLT plus isopropyl alcohol and nonionic surfactants was able to eliminate IBR and BVD viruses within 20 min (29). BVDV was eliminated even without the addition of alcoholic base (30). The QACs were able to inactivate bovine herpes virus 1 (IBR virus) at room temperature but it could not eliminate equine herpesvirus type 1 after 10 min at 0°C at concentrations of 0.05 and 0.02% (w/v). However, the virucidal activity of QACs at room temperature increased with increased duration of exposure and the use of warm water (5).

No studies are available on the effect of QACs or GLT on BRSV although conventional wisdom suggests that they should be highly effective against enveloped viruses. The QACs can solubilize and disrupt lipid envelopes while GLT can cross-link proteins in the envelope (31, 32). In this study, 99.9% of BCV was inactivated within 5 min, which agrees with a previous study in which 0.1% QACs were effective against coronavirus within 15 s (33).

In general, a combination of disinfectants is known to have high efficiency and broad-spectrum action against viruses (33). Environmental factors have a major influence on the disinfection effect including the presence of organic matter, temperature, PH, surface type, and water hardness. These factors were not evaluated in this study although organic matter in the form of horse or bovine serum was present in all virus suspensions.

## Conclusion

The ability of Duplalim to eliminate a wide range of enveloped and non-enveloped viruses may have a direct impact on animal welfare and production. This product could be useful in endemic disease control programs on farms, animal shelters, kennels, and veterinary clinics.

## Data availability statement

The raw data supporting the conclusions of this article will be made available by the authors, without undue reservation.

## Ethics statement

The cell lines used in this study were obtained from American Type Culture Collection (ATCC).

## Author contributions

NS: Methodology, Writing – original draft, Writing – review & editing. AQ-M: Methodology, Visualization, Writing – review & editing. HA: Methodology, Visualization, Writing – review & editing. CY: Methodology, Visualization, Writing – review & editing. GO-B: Conceptualization, Resources, Writing – review & editing. JM-F: Conceptualization, Resources, Writing – review & editing. SG: Conceptualization, Supervision, Writing – review & editing.
